# Polymer Nanodot-Hybridized Alkyl Silicon Oxide Nanostructures for Organic Memory Transistors with Outstanding High-Temperature Operation Stability

**DOI:** 10.1038/srep33863

**Published:** 2016-10-05

**Authors:** Chulyeon Lee, Jooyeok Seo, Jeongnam Kim, Jaehoon Jeong, Hyemi Han, Hwajeong Kim, Youngkyoo Kim

**Affiliations:** 1 Organic Nanoelectronics Laboratory, Department of Chemical Engineering, School of Applied Chemical Engineering, Kyungpook National University, Daegu 41566, Republic of Korea; 2 Priority Research Center, Research Institute of Advanced Energy Technology, Kyungpook National University, Daegu 41566, Republic of Korea

## Abstract

Organic memory devices (OMDs) are becoming more important as a core component in flexible electronics era because of their huge potentials for ultrathin, lightweight and flexible plastic memory modules. In particular, transistor-type OMDs (TOMDs) have been gradually spotlighted due to their structural advantages possessing both memory and driving functions in single devices. Although a variety of TOMDs have been developed by introducing various materials, less attention has been paid to the stable operation at high temperatures. Here we demonstrate that the polymer nanodot-embedded alkyl silicon oxide (ASiO) hybrid materials, which are prepared by sol-gel and thermal cross-linking reactions between poly(4-vinylphenol) (PVP) and vinyltriethoxysilane, can deliver low-voltage (1~5 V) TOMDs with outstanding operation stability (>4700 cycles) at high temperatures (150 °C). The efficient low-voltage memory function is enabled by the embedded PVP nanodots with particular lattice nanostructures, while the high thermal stability is achieved by the cross-linked ASiO network structures.

Transistor-type organic memory devices (TOMDs) have attracted keen interest because of their potentials toward flexible or bendable plastic memory modules^1^,^2^,^3^,^4^,^5^. TOMDs are effective in terms of module (array) configuration because they have both memory and driving (switching) elements in single device, whereas additional transistor circuits are necessary for driving array modules in the case of resistor-type organic memory devices (ROMDs)^6^,^7^,^8^. To date, various organic materials and structures, such as ferroelectric polymers, charge-trapping polymers, polymeric layers with metal nanoparticles and polymer energy well structures, have been employed for TOMDs, but they could not deliver both low-voltage operation and high stability at the same time^9^,^10^,^11^,^12^,^13^,^14^,^15^,^16^,^17^,^18^,^19^,^20^. Very recently, encouraging data retention characteristics at room temperature have been reported for TOMDs with poly(vinyl alcohol) memory gate-insulating layers^21^,^22^.

However, TOMDs with the data retention stability at high temperatures have never been reported so far, even though organic materials (films) are generally understood physically softer and weaker than inorganic materials (films) leading to relatively inferior stability at high temperatures^23^,^24^,^25^. The weak high temperature characteristics of typical organic devices can be attributed to the fact that organic films in devices are made with organic molecules by weak intermolecular (interchain for polymers) interactions such as van der Waals forces, slightly strong hydrogen bonding, etc.^26^,^27^,^28^,^29^. In the case of flash memory devices commercialized with inorganic materials, the guaranteed retention stability (program-erase cycle) reaches 1,000 times and 10,000 times for the triple-level cells (TLCs) and the multi-level cells (MLCs), respectively^30^,^31^,^32^. In addition, such inorganic flash memory devices can be operated at elevated temperatures up to 70 °C^33^,^34^,^35^. Therefore, it is necessary for TOMDs to secure the data retention stability at high temperatures in regard to the positive consideration of commercialization.

In order to achieve the high temperature stability of TOMDs, the core memory layer needs to be thermally stable without loss of memory functions at elevated temperatures. In this regard, organic/inorganic hybrid materials can be one of the effective approaches when it comes to the complementary role of organic and inorganic components in the hybrid materials^36^,^37^,^38^,^39^. In particular, employing polymers as the organic component can be further advantage to the hybrid materials in terms of better stability for flexible TOMDs due to the toughening role of polymers rather than small molecules^40^,^41^,^42^.

In this work, we synthesized novel hybrid nanostructures, featuring polymer nanodot-embedded alkyl silicon oxide (ASiO) networks, by two-step (sol-gel and thermal cross-linking) reactions between poly(4-vinylphenol) (PVP) and triethoxyvinylsilane (or vinyltriethoxysilane - VTES). The resulting cross-linked ASiO hybrids embedded with PVP nanodots (nanoparticles) (X-ASiO-PVP_NP_) were employed as a thermally stable memory layer for the TOMDs with the poly(3-hexylthiophene) (P3HT) channel layers. The hybrid TOMDs could be operated at low voltages (1~5 V) and exhibited outstanding operation stability at high temperatures (150 °C) due to the pronounced hysteresis by the PVP nanodots and the high thermal stability by the cross-linked VTES-derived ASiO network structures.

## Results

### Synthesis and characterizations

**Figure 1:**
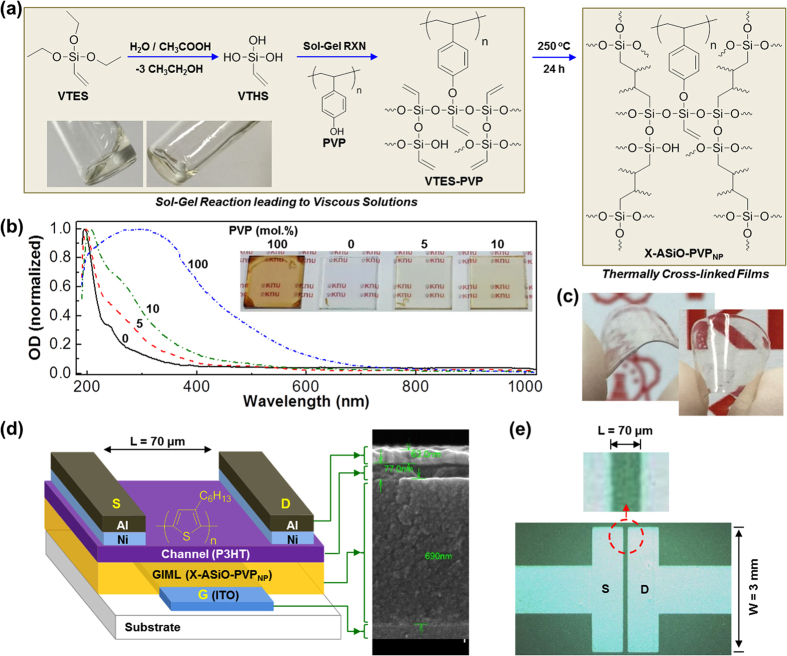
Synthesis of X-ASiO-PVP_NP_ hybrid films and memory device structures. (**a**) Scheme for sol-gel and chemical cross-linking reactions leading to X-ASiO-PVP_NP_ films via the VTES-PVP precursor (see inset photographs) route from VTES and PVP. (**b**) Optical absorption spectra of X-ASiO-PVP_NP_ films coated on ITO-glass substrates according to the molar ratio of PVP (inset: photographs). (**c**) Photographs for the thick X-ASiO-PVP_NP_ films prepared at room temperature. (**d**) Device structure for TOMDs with the P3HT channel layer and the X-ASiO-PVP_NP_ memory gate insulating layer (right: SEM image for the cross-section of device): ‘S’, ‘D’, and ‘G’ denote source, drain, and gate electrodes, respectively. (**e**) Optical microscope image on the channel part of the TOMD in (**d**).

**Figure 2:**
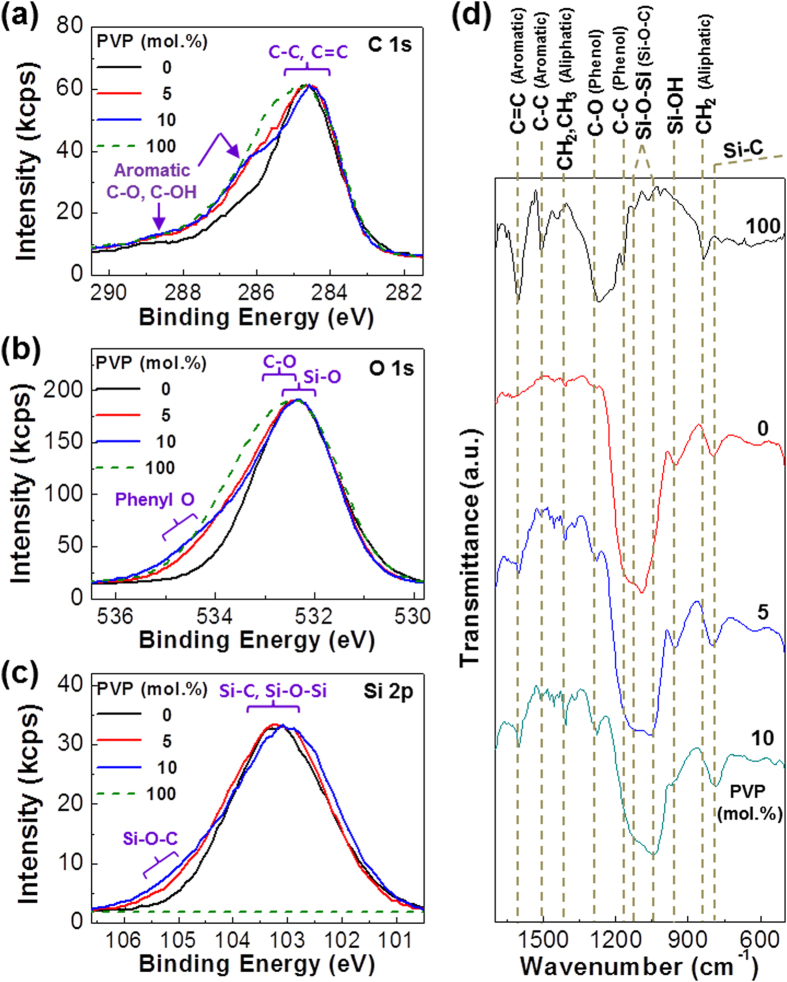
XPS and FT-IR spectra of pristine and X-ASiO-PVP_NP_ hybrid films. (**a**) C1s XPS spectra, (**b**) O1s XPS spectra, (**c**) Si2p XPS spectra, and (**d**) FT-IR spectra. The molar ratio of PVP is given on each graph.

As shown in Fig. 1a, VTES was first hydrolyzed to vinyltetrahydroxysilane (VTHS) in the presence of water and acetic acid, followed by addition of PVP. As soon as VTES is converted to VTHS, sol-gel reactions are considered to begin among VTHS molecules as well as between VTHS and PVP (Si-OH groups in VTHS and C-OH groups in PVP), leading to the precursor sol solutions (VTES- PVP). The precursor solutions were coated on quartz substrates or indium-tin oxide (ITO)-coated glass substrates leading to the VTES-PVP precursor films, which were finally converted to the cross-linked hybrid (X-ASiO-PVP_NP_) films via thermal curing reaction between double bonds in the silicon atoms. The optical measurement disclosed that the optical absorption spectra (absorption edge) of hybrid (X-ASiO-PVP_NP_) films were gradually red-shifted as the PVP content increased (see Fig. 1b). However, the optical transparency of films was well maintained even though the film color was changed to slightly yellowish (see the inset photographs in Fig. 1b and Fig. S1). Here we note that the precursor films before thermal cross-linking reactions could be as thick as more than 200 μm by drop-casting from their precursor solutions (see Fig. 1c). The formation of Si-O-C bonds, which are caused by the reaction between the hydroxyl (C-OH) groups in the PVP chains and the silanol (Si-OH) groups in the VTHS domains during sol-gel reactions, was proven by the X-ray photoelectron spectroscopy (XPS) and Fourier Transform-Infrared (FT-IR) spectroscopy measurements (see Fig. 2). As shown in Fig. 1d, the cross-linked hybrid (X-ASiO-PVP_NP_) films were well coated on the patterned ITO-glass substrates so as to make a gate-insulating memory layer that is placed beneath the P3HT channel layer in the hybrid TOMDs (see the optical microscope images for the channel area in Fig. 1e).

In order to verify whether the hydroxyl (C-OH) groups in the PVP chains were certainly reacted with the silanol (Si-OH) groups in the VTHS domains during sol-gel reactions, X-ray photoelectron spectroscopy (XPS) was employed to measure the evolution of Si-O-C_Phenyl_ bonds. As shown in Fig. 2a, the C1s peaks (shoulders) at ca. 286.5 eV and 288.4 eV were emerged as the PVP content increased, which can be ascribed to the aromatic C-OH because the XPS intensity at this energy range was ignorable for the pristine ASiO films without PVP. Similarly, the O1s shoulders at around 535 eV (aromatic C-O) became pronounced with the PVP content though no XPS signal was measured at this region for the pristine ASiO films. These results confirm the presence of PVP in the hybrid films. Finally, the Si2p peaks revealed the formation of Si-O-C_Phenyl_ bonds because the shoulders between 105 eV and 106 eV were gradually increased with the PVP content compared to no signal for the pristine PVP films (note that the Si-O-C_Ethyl_ bonds were almost completely removed during hydrolysis reaction). The evolution of Si-O-Si and Si-O-C_Phenyl_ bonds was measured at the wavenumber range of 900~1200 cm^−1^ from the Fourier Transform-Infrared (FT-IR) spectra (Fig. 2d)^43^, which also delivered the gradual increase in the aromatic C-C and C = C peaks with respect to the PVP content^44^.

### Nanostructures

**Figure 3:**
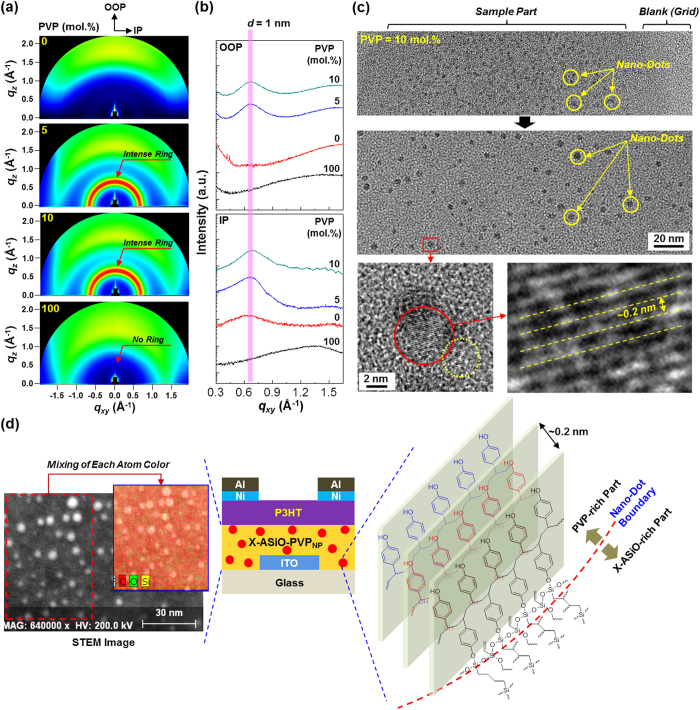
Nanostructures of pristine and X-ASiO-PVP_NP_ hybrid films. (**a**) 2D GIXD images, (**b**) 1D GIXD profiles in the out-of-plane (OOP) and in-plane (IP) directions, (**c**) HRTEM images for the X-ASiO-PVP_NP_ hybrid film (10 mol.% PVP), and (**d**) STEM images for the X-ASiO-PVP_NP_ hybrid film (10 mol.% PVP) and illustration for the ordered structure of PVP chains inside the nanodots.

The nanostructures of the cross-linked hybrid (X-ASiO-PVP_NP_) films were first investigated by employing synchrotron radiation grazing incidence X-ray diffraction (GIXD) techniques. As shown in the 2D GIXD images (Fig. 3a), very weak (almost unrecognizable) Debye diffraction ring was measured for the pristine ASiO films (PVP content = 0 wt.%), while no particular diffractions were measured for the pristine PVP films. Interestingly, an intense Debye ring was measured for the cross-linked hybrid (X-ASiO-PVP_NP_) films with 5 mol.% PVP. The similar strong Debye ring was also measured for the hybrid films with 10 mol.% PVP. Based on the scattering vectors for the Debye rings, the d-spacing (*d*) values calculated were in the range of 0.62~1.57 nm. The detailed investigation with the 1D GIXD profiles (Fig. 3b) disclosed that the intense Debye rings have a maximum peak at around *q*_xy_ = 0.66 Å^−1^ (2θ = 6.75°), leading to *d* = 1 nm, in the directions of both out-of-plane (OOP) and in-plane (IP). Considering the GIXD results, it is shortly concluded that the addition of PVP had a strong influence on the reorganization (recrystallization) of VTES molecules.

Both high-resolution and scanning transmission electron microscopy (HRTEM and STEM) measurements were performed to further understand the nanostructure of the cross-linked hybrid films. As shown in Fig. 3c, the HRTEM measurements revealed the existence of very small nanodots that are randomly distributed with various sizes (1~3 nm) in the cross-linked hybrid (X-ASiO-PVP_NP_) films (see also Fig. S2a,b). Further HRTEM measurement with high magnification showed that the small nanodots consist of a particular lattice nanostructure with an inter-lattice spacing of ca. 0.15~0.2 nm, indicative of highly ordered states (see the bottom images in Fig. 3c). Interestingly, as shown in Fig. 3d, the nanodots were identified as the PVP domains by the STEM measurement through the composition analysis (see the focused area in Fig. S2c). Because the nanodots exhibited dark spots in the HRTEM images and bright spots in the STEM images, they are confirmed a well-ordered phase that is sufficient to make proper electron diffractions^45^. Taking into account the TEM analysis results, it is supposed that the nanodots consist of well-ordered PVP chains as illustrated in Fig. 3d (right). This well-ordered state could be made via nanoscale-phase separation processes during sol-gel and thermal cross-linking reactions because of the increased viscosity leading to the different surface energy between the PVP phase and the ASiO precursor sol phase.

### Hysteresis and memory mechanism

**Figure 4:**
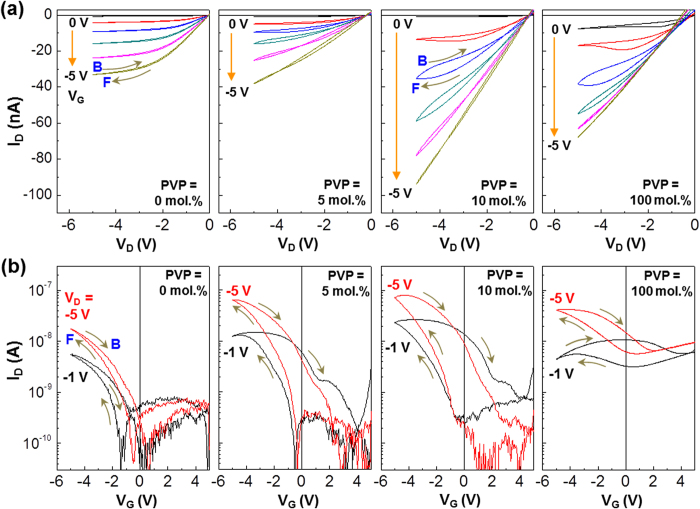
Transistor characteristics and hysteresis of devices. Output (**a**) and transfer (**b**) characteristics for the TOMDs with the pristine and/or X-ASiO-PVP_NP_ hybrid gate insulating layers. ‘F’ and ‘B’ represent forward (V_D_: from 0 V to −5 V in (**a**); V_G_: from +5 V to −5 V in (**b**)) and backward (V_D_: from −5 V to 0 V in (**a**); V_G_: from −5 V to +5 V in (**b**)) sweeps, respectively.

Next, the cross-linked hybrid (X-ASiO-PVP_NP_) films were examined as a gate-insulating layer for the transistor structure as illustrated in Fig. 1d. As shown in the output curves (Fig. 4a), all devices exhibited typical p-type transistor characteristics and could be operated at low voltages (−1~−5 V in absolute value). Interestingly, a hysteresis was measured in the output curves for the devices with the X-ASiO-PVP_NP_ layers, which became more pronounced as the content of PVP increased. In addition, the drain current (I_D_) at the same voltage condition was higher for the devices with the X-ASiO-PVP_NP_ layers (10 mol.% PVP) than those with the pristine X-ASiO layers or the pristine PVP layers. Note that the devices with the pristine PVP layers showed high leakage currents even at zero gate voltage (V_G_ = 0 V). As observed from the transfer curves in Fig. 4b, the current hysteresis between forward and backward sweeps in the devices with the pristine X-ASiO layers was quite small and not improved even by increasing the drain voltage (V_D_) from −1 V to −5 V. However, the hysteresis became certainly pronounced when 5 mol.% PVP was added. Further addition of PVP (10 mol.%) led to more improved hysteresis in the transfer curves (see Fig. S3 for the gradual hysteresis change with the drain voltage). In addition, the on/off ratio at V_D_ = −5 V was noticeably improved from 1.2 × 10^3^ (0 mol.%) to 1.1 × 10^4^ (5 mol.%) and 4.4 × 10^5^ (10 mol.%) by addition of PVP. The PVP addition contributed to the improved hole mobility from 2.89 × 10^−3^ (0 mol.%) to 2.28 × 10^−2^ (5 mol.%) and 1.3 × 10^−1^ (10 mol.%) (see Fig. S4).

**Figure 5:**
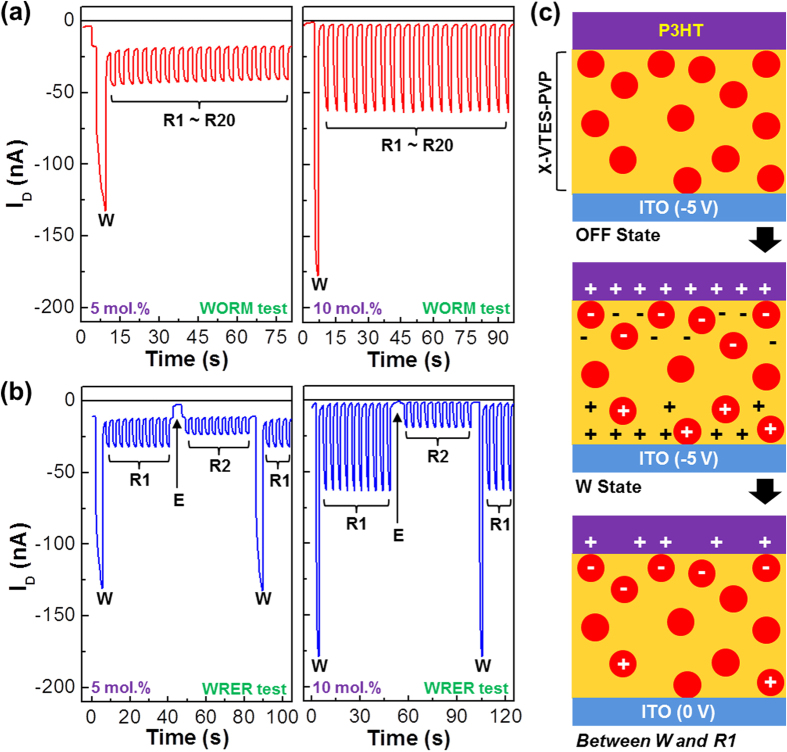
Memory characteristics and mechanism. (**a**) Writing-once-reading-many (WORM) operations for the TOMDs with the X-ASiO-PVP_NP_ hybrid memory gate insulating layers [writing (W): V_G_ = −5 V and V_D _= −3 V for 3 s; many reading (R1~R20): V_G_ = −1 V and V_D_ = −3 V for 3 s]. (**b**) Writing-reading-erasing-reading (WRER) operations for the TOMDs with the X-ASiO-PVP_NP_ hybrid memory gate insulating layers [writing (W): V_G_ = −5 V and V_D_ = −3 V for 3 s; reading-1 (R1): V_G_ = −1 V and V_D_ = −3 V for 3 s; erasing (E): V_G_ = +5 V and V_D_ = −3 V for 3 s; reading-2 (R2): V_G_ = −1 V and V_D_ = −3 V for 3 s]. (**c**) Illustration for the operation mechanism of the present TOMDs with the X-ASiO-PVP_NP_ hybrid memory gate insulating layers.

Based on the hysteresis characteristics, memory operation tests were performed for the devices with the X-ASiO-PVP_NP_ layers. The writing-once-reading-many (WORM) test showed that both 5 mol.% and 10 mol.% PVP devices properly made proper operations of reading (V_G _= −1 V and V_D _= −3 V) after writing (V_G _= −5 V and V_D _= −3 V) (see Fig. 5a). However, the 10 mol.% PVP devices showed better WORM characteristics with stable signals. In the case of writing-reading-erasing-reading (WRER) cycle test (Fig. 5b), the 10 mol.% devices exhibited more stable memory functions with clearer drain current difference between reading-1 (R1) after writing (W) and reading-2 (R2) after erasing (E). In contrast, the 5 mol.% devices showed less stable WRER characteristics in the presence of small drain current difference. The superior memory performance of the 10 mol.% PVP devices is also evidenced from the repeated WORM and WRER operations (see Fig. S5). Hence the 10 mol.% PVP devices can be called one of TOMDs with proper memory functions.

Taking into account the nanostructures measured in Fig. 3, the charge traps in the PVP_NP_ parts of the X-ASiO-PVP_NP_ layers can be proposed as a core factor for memory operation for the present hybrid TOMDs. As illustrated in Fig. 5c, the PVP_NP_ parts are charged simultaneously when the X-ASiO-PVP_NP_ layers undergo polarization upon applying bias between the gate and source electrodes (V_G_ = −5 V). However, the charges made in the PVP_NP_ parts cannot easily go away but trapped inside the PVP_NP_ parts by the heterogeneous dielectric interfaces between PVP_NP_ and X-ASiO because of different nature of materials. So the charged PVP_NP_ parts play a critical role in delivering the hysteresis characteristics of the hybrid TOMDs.

### High-temperature stability

**Figure 6:**
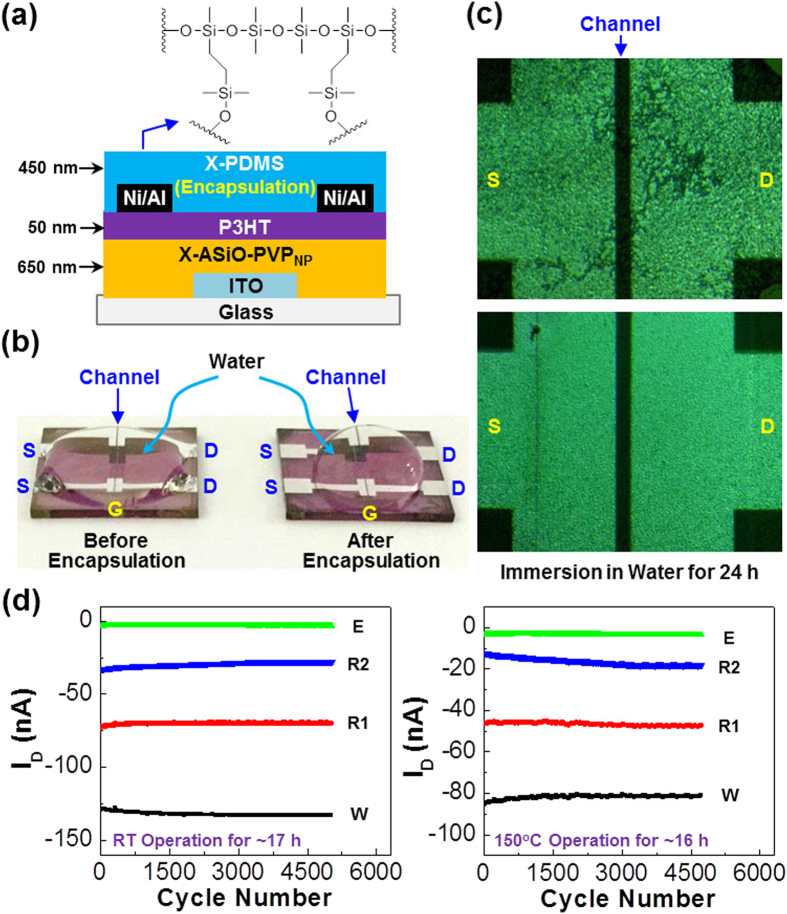
Encapsulation of devices and high-temperature retention characteristics. (**a**) Illustration for the TOMDs with the cross-linked PDMS (X-PDMS) layers. (**b**) Photographs for water drop test on the channel part of devices: (left) bare device (without encapsulation), (right) encapsulated device. (**c**) Optical microscope images for the channel part of devices after water drop test: (top) bare device (without encapsulation), (bottom) encapsulated device. (**d**) Writing-reading-erasing-reading (WRER) operations for the encapsulated TOMDs with the X-ASiO-PVP_NP_ hybrid memory gate insulating layers at room temperature (left) and 150 °C (right) [writing (W): V_G_ = −5 V and V_D_ = −3 V for 3 s; reading-1 (R1): V_G_ = −1 V and V_D_ = −3 V for 3 s; erasing (E): V_G_ = +5 V and V_D_ = −3 V for 3 s; reading-2 (R2): V_G_ = −1 V and V_D_ = −3 V for 3 s].

Considering the outstanding WORM and WRER characteristics, the TOMDs with the X-ASiO-PVP_NP_ layers (PVP = 10 mol.%) were chosen for the data retention test, which is usually performed by repeating the WRER operation over several thousand cycles^46^,^47^,^48^. Prior to the retention test, the TOMDs were encapsulated with the cross-linked poly(dimethylsiloxane) (PDMS) layers (see Fig. 6a and the method section for the detailed process). As shown in Fig. 6b, water drops were well spread over the channel area in the case of the bare devices without any encapsulation, which implies good interactions between Al electrodes and water molecules. However, the devices encapsulated with the cross-linked PDMS layers showed a restricted spreading of water drop. Next, both devices were immersed into water for 24 h in order to examine the performance of encapsulation. As displayed in Fig. 6c, the Al electrodes in the channel area were safe without any defects in the case of the encapsulated devices, whereas the Al electrode parts were seriously damaged for the bare devices (without encapsulation).

Hence the 10 mol.% PVP devices, which were encapsulated with the cross-linked PDMS layers, were used for the measurement of retention characteristics and high temperature operations. As shown in Fig. 6d (left), the encapsulated devices exhibited very stable retention characteristics up to 5000 WRER cycles. The deviation of reading-1 (R1) current was only 4.3% after 5000 cycles, while that of reading-2 (R2) after erasing was 15.5% compared to the initial value. Next, the encapsulated devices were loaded on a hot stage in order to examine the retention characteristics at 150 °C. As shown in Fig. 6d (right), the drain current deviation after 4750 cycles at 150 °C was 1.5% and 44% for reading-1 (R1) and reading-2 (R2), respectively. Here we note that the drain current level at 150 °C was slightly changed from that at room temperature, which can be attributable to the thermal effect on charge transport (mobility) as well as the different contact environment between the Al electrodes and the probes in the measurement system. Here it is also worthy to note that the P3HT channel layers are also strong enough to withstand the thermal shock at 150 °C because the present P3HT polymers with high regioregularity (96%) possess relatively high glass transition temperatures, close to but lower than the melting points (210~240 °C), due to the very small portion of amorphous (regiorandom) parts (4%)^49^,^50^,^51^. In contrast to the outstanding retention characteristics of the encapsulated devices at such high temperature (150 °C), a catastrophic current fluctuation was measured for the bare devices at 150 °C and even at room temperature (see Fig. S6). It is also worthy to note that the pristine PVP devices (PVP = 100 mol.%) exhibited much poorer hysteresis at 150 °C than room temperature, indicative of an impossible state for memory operation (see Fig. S7). Hence this result implies that the present encapsulated devices with the X-ASiO-PVP_NP_ layers (PVP = 10 mol.%), hybrid TOMDs, can be used as a durable memory device for high temperature applications such as security camera built in car, control systems for fire-fighters and steel mill industries, rescue robots for nuclear power stations, memory systems for space shuttles, etc.

## Discussion

In conclusion, novel polymer nanodot-embedded alkyl silicon oxide hybrids, X-ASiO-PVP_NP_, were successfully prepared via sol-gel and chemical cross-linking reactions. The surface of PVP nanodots (PVP_NP _= 1~3 nm from HRTEM) with a particular lattice nanostructure (inter-lattice spacing = 0.15~0.2 nm) was found to make covalent bonds with the cross-linked alkyl silicon oxide (X-ASiO), which might be a driving force to generate such a small PVP_NP_ leading to high thermal stability. The hybrid transistor-type organic memory devices (TOMDs) with the X-ASiO-PVP_NP_ layers exhibited stable operation at 150 °C during >4750 WRER cycles, which is the first record for TOMDs tested at a high temperature. Therefore, the present X-ASiO-PVP_NP_ hybrid materials are expected to be a landmarking milestone for achieving durable organic memory devices with excellent operation stability at high temperatures. In addition, the synthesis protocols of X-ASiO-PVP_NP_ materials can be useful to invent super-strong flexible transparent substrates and thermo-resistive functional coatings for flexible electronic devices because the embedded (hybridized) polymer nanodots are expected to play a critical role in compensating mechanical stresses together with high thermal stability by the X-ASiO domains.

## Methods

### Hybrid solutions via sol-gel reactions

The hybrid solutions were prepared via sol-gel reactions of vinyl triethoxysilane (VTES) and poly(4-vinylphenol) (PVP, average molecular weight = 25 kDa), which were purchased from Sigma-Aldrich Co. (St Louis, Mo, USA), in the presence of deionized (DI) water and acetic acid (Sigma-Aldrich). The composition of PVP to VTES was 0, 5, and 10 mol. %, while the molar ratio of DI water and acetic acid was fixed at 1:1:6. The mixture solutions, which contain VTES, PVP, DI water and acetic acid, were subject to vigorous stirring initially, followed by sol-gel reactions at room temperature for 24 h.

### Fabrication of hybrid memory transistors

Indium-tin oxide (ITO)-coated glass substrates (sheet resistance = 10 Ω/cm^2^) were subject to a photolithography process to make the ITO patterns with a 1 × 12 mm stripe for gate (G) electrodes. After cleaning the patterned ITO-glass susbtrates in an ultrasonic bath with acetone and isopropyl alcohol (30 min), the clenaed ITO-glass substrates were treated with UV-ozone (28 mW/cm^2^ for 20 min) by utilizing UV-ozone cleaner (UVO cleaner, Ahtech LTS Co., Ltd) in order to remove any organic contaminants remained. Next, the VTES-PVP precursor films were spin-coated on the ITO-glass substrates, followed by thermal curing processes at 250 °C for 6 h leading to chemically cross-linked hybrid (X-ASiO-PVP_NP_) films (thickness = 700 nm). On top of the X-ASiO-PVP_NP_ layers, the P3HT channel layers (thickness = 60 nm) were spin-coated at 1500 rpm for 30 s by using the P3HT solutions, which were prepared by dissolving the P3HT polymer (weight-average molecular weight = 70 kDa, polydispersity index = 1.8, regioregularity = 96%, Rieke Metals) in toluene at a solid concentration of 13 mg/ml. The spin-coated P3HT channel layers were soft-baked at 70 °C for 15 min and transferred to a vacuum chamber installed inside a nitrogen-filled glove box system. Finally, the source (S) and drain (D) electrodes were formed by successively depositing nickel (Ni, thickness = 15 nm) and aluminum (Al, thickness = 60 nm) electrodes on the P3HT layers through a shadow mask. The channel length and width of devices were 70 μm and 2 mm, respectively (see Fig. 1d). The fabricated devices were encapsulated by the cross-linked poly(dimethyl siloxane) (PDMS) layers (Sylgard 184, 10 g clip-pack, Sigma-Aldrich Co., St Louis, Mo, USA, thickness = 450 nm), which were cured at room temperature. We note that the X-ASiO-PVP_NP_ films were prepared on quartz substrates for the measurement of optical absorption spectra but indium-tin oxide (ITO)-coated glass substrates were used for other measurements including XPS spectra, FT-IR spectra and GIXD images.

### Measurements

The thickness of films was measured using a surface profiler (Alpha Step 200, Tencor). An ultraviolet-visible absorption spectrometer (Optizen 2120UV, Mecasys) was used for the measurement of optical absorption spectra of films. The cross-sections of hybrid memory transistors were examined with a field-emission scanning electron microscope (FESEM, S-4800, Hitachi), while an optical microscope (SV-55, Sometech) was used to inspect the surface of hybrid films and the top part of devices. The memory transistor charateristics of devices were measured using a semiconductor parameter analyzers (Keithley 4200, Keithley 2636B, Keithley Instruments Inc.), which are connected to a sample holder system with heating units inside a glove-box probe station (PS-CPSN2, Modu-Systems). The XPS spectra of hybrid films were measured with a X-Ray photoelectron spectrometer (ESCALAB 250, Thermo Scientific, Inc.), while a Fourier transform-infrared spectrometer (FT-IR, 5700 Continum, Thermo Scientific, Inc.) with the attenuated total reflection (ATR) mode was used for the measurement of functional groups in the hybrid film samples. The nanostructure of hybrid films was measured using a synchrotron radiation grazing incidence angle X-ray diffraction system (GIXD, wavelength = 1.212969 Å, Pohang Accelerator Laboratory).

## Additional Information

**How to cite this article**: Lee, C. *et al*. Polymer Nanodot-Hybridized Alkyl Silicon Oxide Nanostructures for Organic Memory Transistors with Outstanding High-Temperature Operation Stability. *Sci. Rep.* **6**, 33863; doi: 10.1038/srep33863 (2016).

## Supplementary Material

Supplementary Information
