# Supplementary material for: Polymer Nanodot-Hybridized Alkyl Silicon Oxide Nanostructures for Organic Memory Transistors with Outstanding High-Temperature Operation Stability
Source: Sci Rep. 2016 Oct 5;6:33863. doi: 10.1038/srep33863 (PMC5050446; doi:10.1038/srep33863)
Supplement: Supplementary Information [file srep33863-s1.pdf]

<Supplementary Information>

Polymer Nanodot-Hybridized Alkyl Silicon Oxide Nanostructures for Organic Memory Transistors with Outstanding High-Temperature Operation Stability

Chulyeon Lee[1], Jooyeok Seo[1], Jeongnam Kim[1], Jaehoon Jeong[1], Hyemi Han[1], Hwajeong Kim[1,2,]*, Youngkyoo Kim[1,]*

[1]*Organic Nanoelectronics Laboratory, Department of Chemical Engineering, School of Applied Chemical Engineering, Kyungpook National University, Daegu 41566, Republic of Korea*

[2]*Priority Research Center, Research Institute of Advanced Energy Technology, Kyungpook National University, Daegu 41566, Republic of Korea*

*Corresponding Authors: Prof. Y. Kim

(Email) ykimm@knu.ac.kr

(Tel) +82-53-950-5616

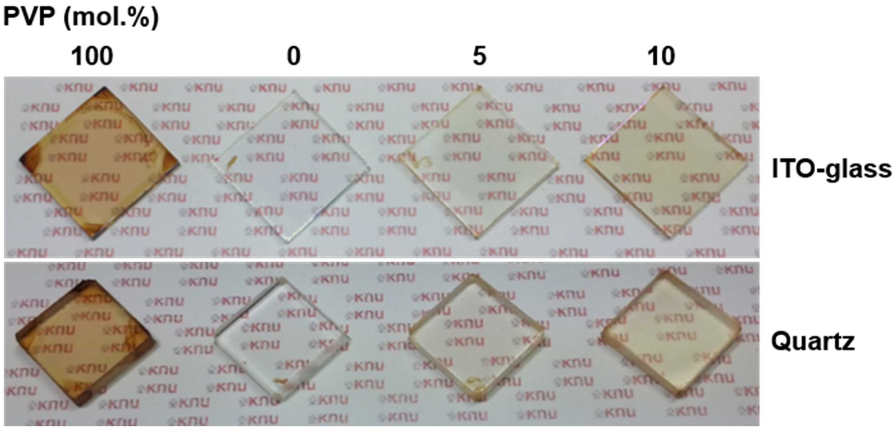

Figure S1. Color change of X-ASiO-PVP$_{NP}$ films coated on ITO-glass (top) and quartz (bottom) substrates according to the molar ratio of PVP (left: pristine PVP films).

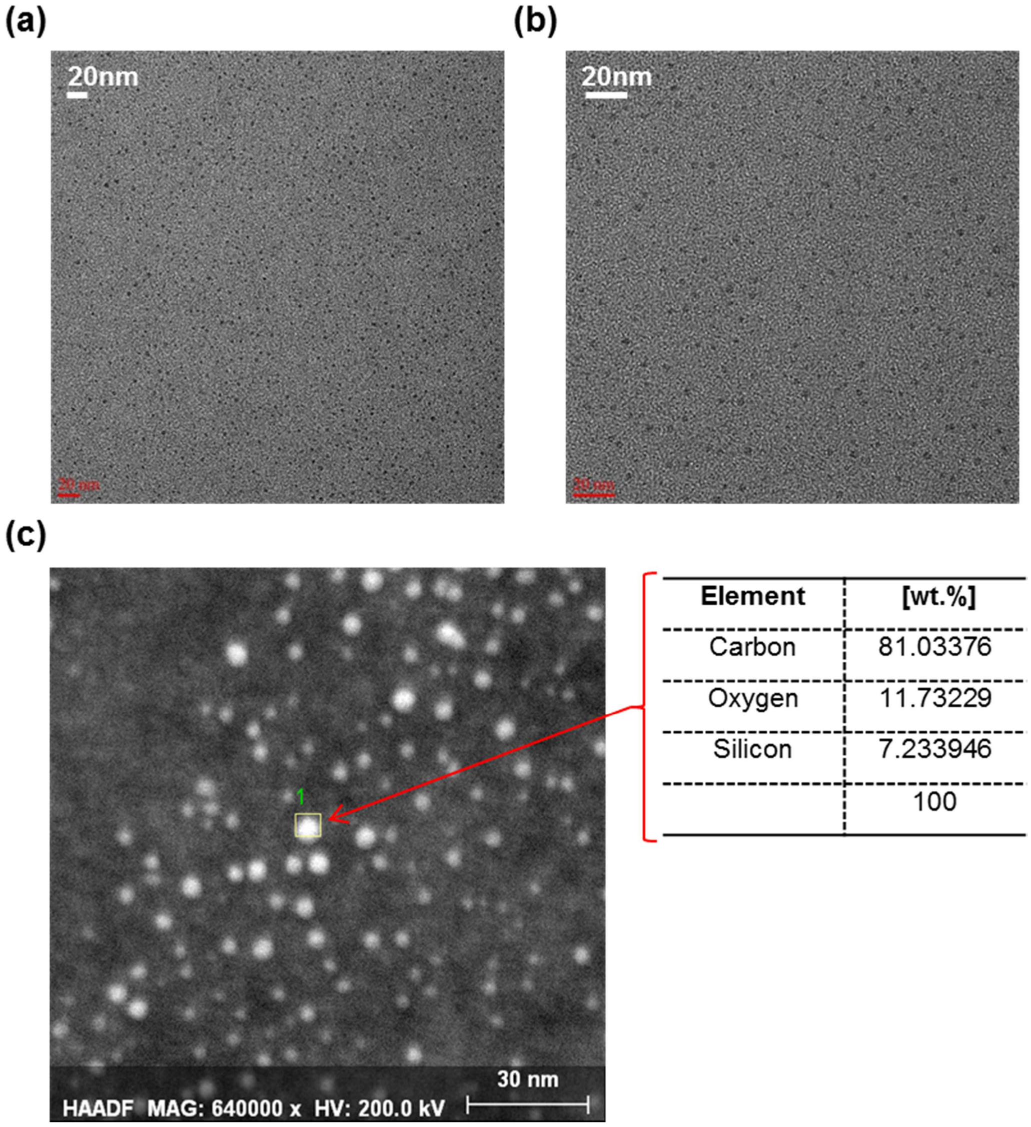

Figure S2. (a,b) HRTEM images for two different X-ASiO-PVP$_{NP}$ hybrid films (10 mol.% PVP) prepared on different ITO-glass substrates. (c) STEM image for the X-ASiO-PVP$_{NP}$ hybrid film and the element analysis result (see Table) on the nanodot marked as 1 (see the yellow square part pointed by the red arrow).

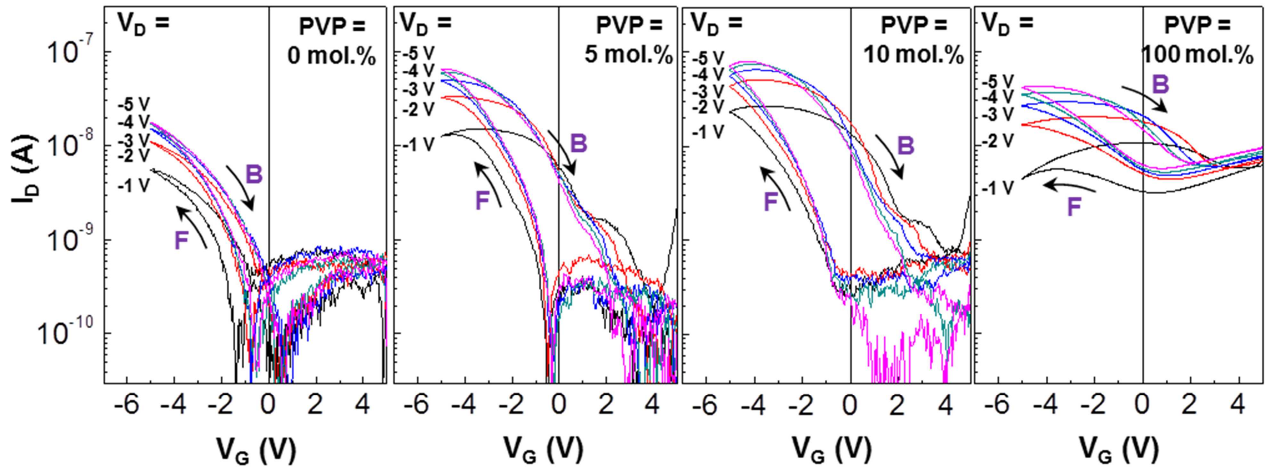

Figure S3. Transfer curves for the TOMDs with the pristine (PVP and X-ASiO) and hybrid (X-ASiO-PVP$_{NP}$) gate insulating layers according to the drain voltage (V_D). The gate voltage (V_G) was scanned from +5 V to -5 V for the forward (F) sweep, while V_G was changed from -5 V to +5 V for the backward (B) sweep.

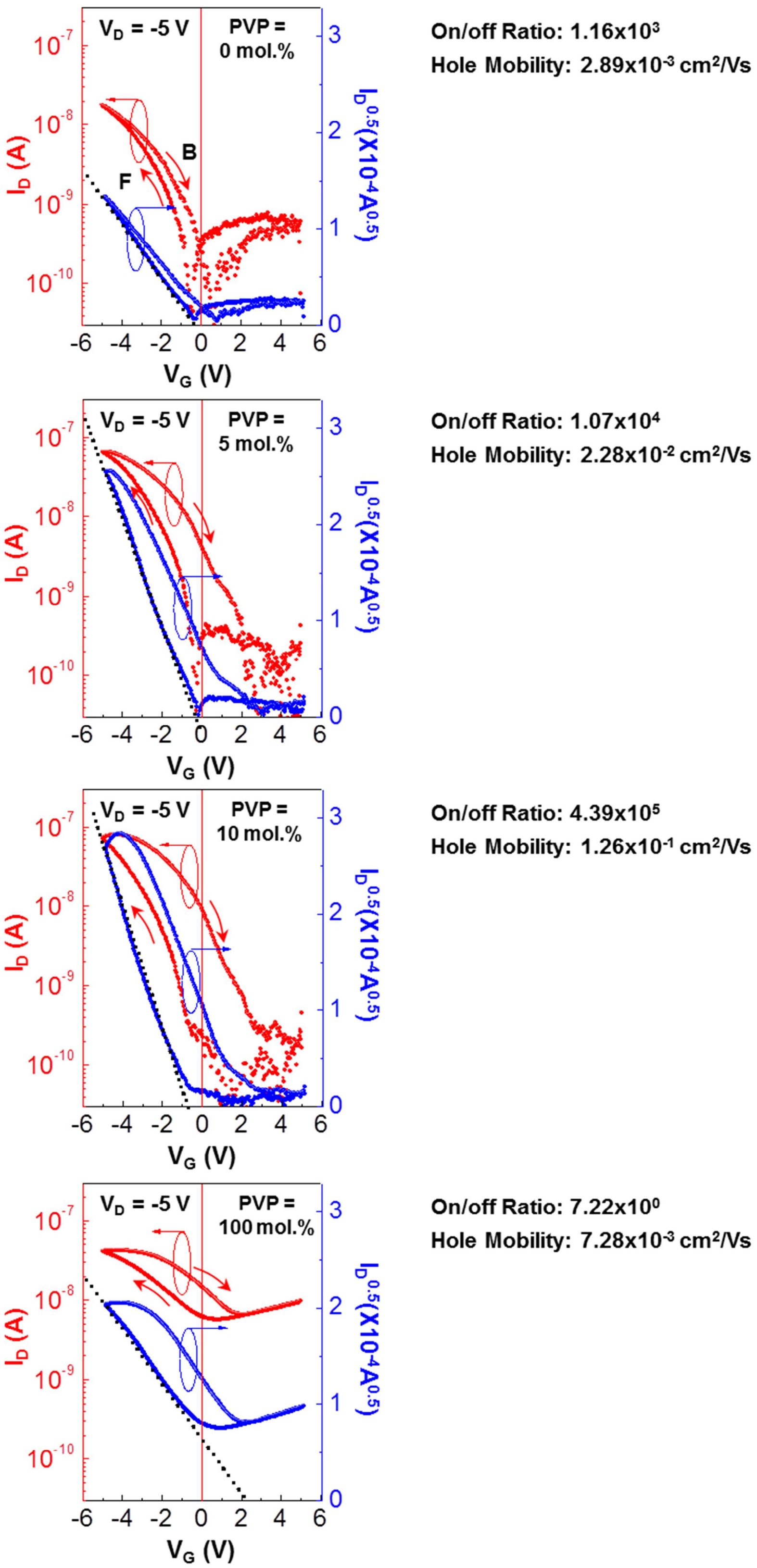

Figure S4. Transfer curves (red) and $I_D^{0.5}$-V_G plots (blue) for the TOMDs with the pristine (PVP and X-ASiO) and hybrid (X-ASiO-PVP$_{NP}$) gate insulating layers at V_D = -5 V. The gate voltage (V_G) was scanned from +5 V to -5 V for the forward (F) sweep, while V_G was changed from -5 V to +5 V for the backward (B) sweep.

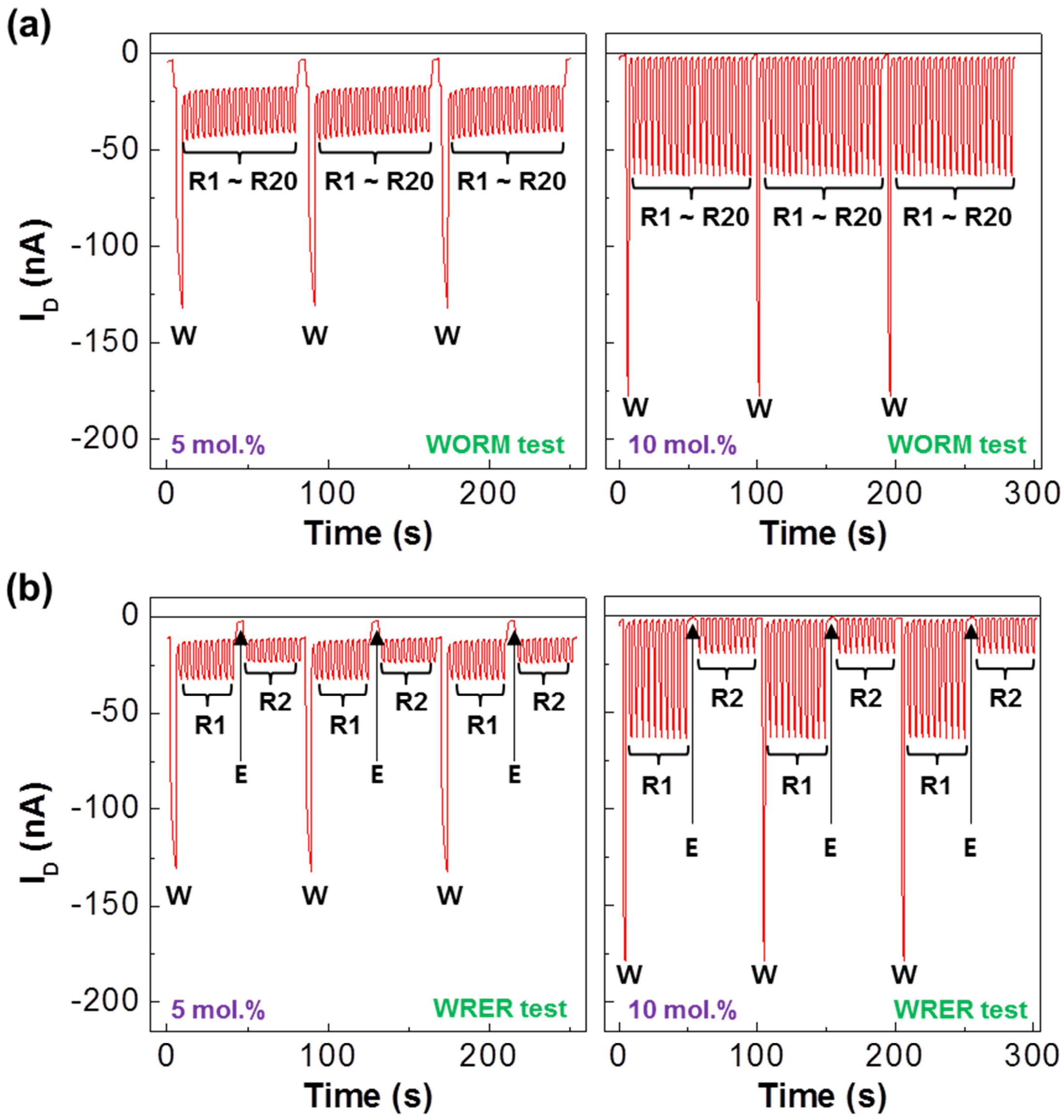

Figure S5. (a) Writing-once-reading-many (WORM) operations for the TOMDs with the X-ASiO-PVP$_{NP}$ hybrid memory gate insulating layers [writing (W): V_G = -5 V and V_D = -3 V for 3 s; many reading (R1~R20): V_G = -1 V and V_D = -3 V for 3 s]. (b) Writing-reading-erasing-reading (WRER) operations for the TOMDs with the X-ASiO-PVP$_{NP}$ hybrid memory gate insulating layers [writing (W): V_G = -5 V and V_D = -3 V for 3 s; reading-1 (R1): V_G = -1 V and V_D = -3 V for 3 s; erasing (E): V_G = +5 V and V_D = -3 V for 3 s; reading-2 (R2): V_G = -1 V and V_D = -3 V for 3 s].

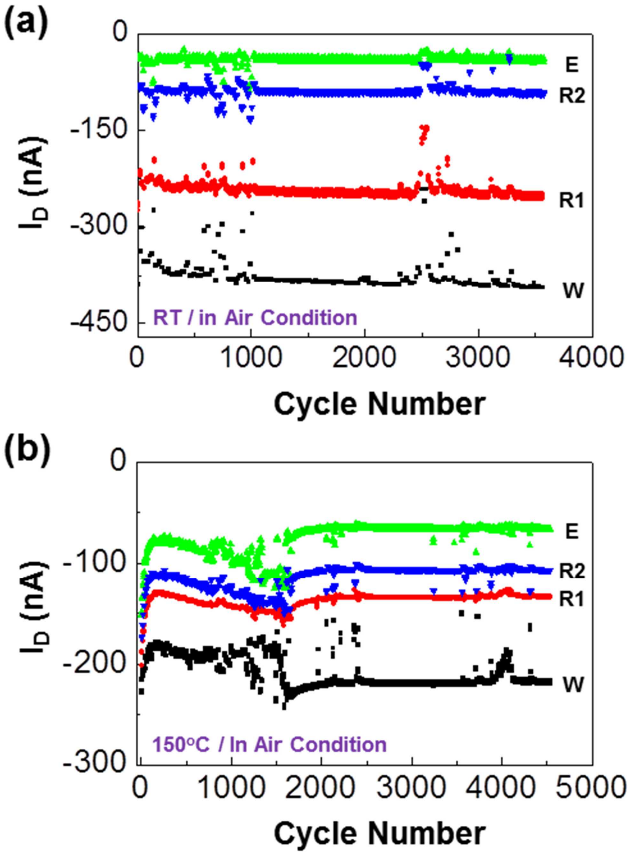

Figure S6. Writing-reading-erasing-reading (WRER) operations in air ambient condition for the TOMDs with the X-ASiO-PVP$_{NP}$ hybrid memory gate insulating layers at room temperature (a) and 150 ^{o}C (b) [writing (W): V_G = -5 V and V_D = -3 V for 3 s; reading-1 (R1): V_G = -1 V and V_D = -3 V for 3 s; erasing (E): V_G = +5 V and V_D = -3 V for 3 s; reading-2 (R2): V_G = -1 V and V_D = -3 V for 3 s].

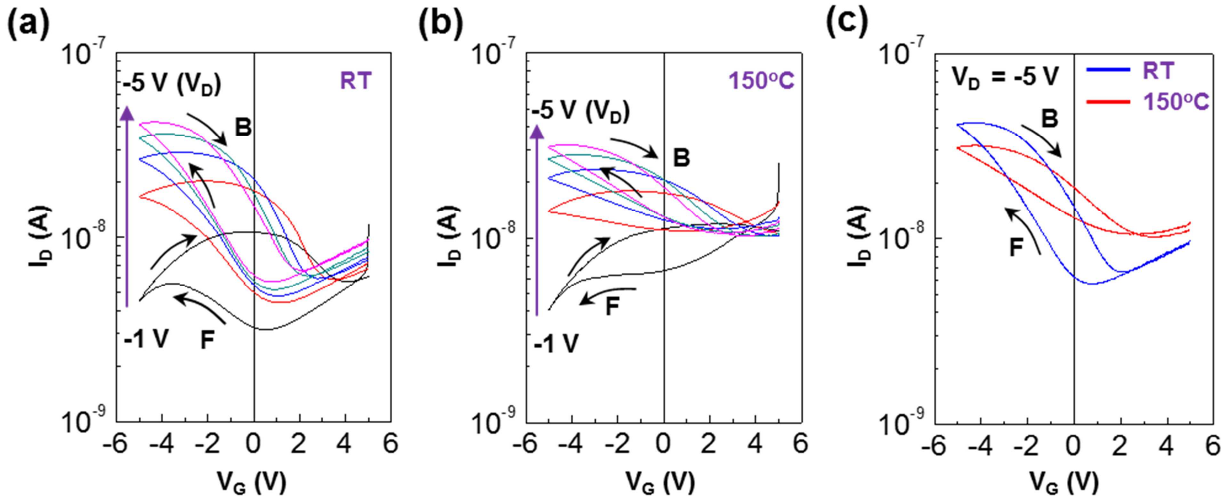

Figure S7. Transfer curves for the devices with the pristine PVP gate insulating layers operated at room temperature (a) and 150 °C (b). (c) Comparison of transfer curves at V_D = -5 V taken from (a) and (b). The gate voltage (V_G) was scanned from +5 V to -5 V for the forward (F) sweep, while V_G was reversely scanned from -5 V to +5 V for the backward (B) sweep. Note that the present pristine PVP devices exhibited poor (drain current) hysteresis at room temperature and the hyteresis became even worse at 150 °C leading to insufficient states for memory operation.
